# Retrospective observational study evaluating zinc plasma level in patients undergoing thoracoabdominal aortic aneurysm repair and its correlation with outcome

**DOI:** 10.1038/s41598-021-03877-6

**Published:** 2021-12-21

**Authors:** Benjamin Rolles, Inga Wessels, Panagiotis Doukas, Drosos Kotelis, Lothar Rink, Margherita Vieri, Fabian Beier, Michael Jacobs, Alexander Gombert

**Affiliations:** 1 Department of Hematology, Oncology, Hemostaseology and Stem Cell Transplantation, Faculty of Medicine, RWTH Aachen University, Pauwelsstrasse 30, 52074 Aachen, Germany; 2 Center for Integrated Oncology, Aachen Bonn Cologne Duesseldorf (CIO ABCD), Cologne, Germany; 3 Institute of Immunology, Faculty of Medicine, RWTH Aachen University, Pauwelsstr. 30, 52074 Aachen, Germany; 4 Department of Vascular Surgery, European Vascular Center Aachen-Maastricht, University Hospital RWTH Aachen, Aachen, Germany

**Keywords:** Biomarkers, Outcomes research

## Abstract

Thoracoabdominal aortic aneurysm (TAAA) repair is related to a relevant morbidity and in-hospital mortality rate. In this retrospective observational single-center study including serum zinc levels of 33 patients we investigated the relationship between zinc and patients’ outcome following TAAA repair. Six patients died during the hospital stay (18%). These patients showed significantly decreased zinc levels before the intervention (zinc levels before intervention: 60.09 µg/dl [survivors] vs. 45.92 µg/dl [non-survivors]). The post-interventional intensive care SOFA-score (Sepsis-related organ failure assessment) (at day 2) as well as the SAPS (Simplified Acute Physiology Score) (at day 2) showed higher score points in case of low pre-interventional zinc levels. No significant correlation between patient comorbidities and zinc level before intervention, except for peripheral arterial disease (PAD), which was significantly correlated to reduced baseline zinc levels, was observed. Septic shock, pneumonia and urinary tract infections were not associated to reduced zinc levels preoperatively as well as during therapy. Patients with adverse outcome after TAAA repair showed reduced pre-interventional zinc levels. We speculate that decreased zinc levels before intervention may be related to a poorer outcome because of poorer physical status as well as negatively altered perioperative inflammatory response.

## Introduction

Treatment of thoracoabdominal aortic aneurysms (TAAA), either by open or endovascular procedures, is related to a high risk of postoperative complications and in-hospital mortality^1^. Careful planning of the surgical approach is of utmost importance, and it requires diligent assessment of the clinical condition of the patient and the comorbidities. A reliable prognostic biomarker could be of high value in this context leading to improved preoperative care^2^. Next to familiar predisposition, age, gender and genetic factors, atherosclerosis is the most common causality for the pathogenesis of TAAA^3^. Atherosclerotic diseases are generally associated with increased systemic inflammation^4^ and therefore the patients’ zinc status, as a biomarker directly connected to the immune response, was examined in this study^5^. Zinc, as an essential trace element, has multiple functions such as in wound healing and the function of the immune system^6^. In humans, an overdose of zinc is rare, whereas its deficiency is a frequent state, especially in elderly people and in patients suffering from chronic diseases^7,8^. A reduced dietary intake of zinc may be one of the most common causes of deficiency^9^. One of the first symptoms of zinc deficiency is the impairment of immune function leading to an excessive release of proinflammatory cytokines and an increased susceptibility towards infections^10^. In developing countries, zinc deficiency is the fifth most common cause of increased overall disease burden, measured in disability-adjusted life years (DALYs)^11^. Physiologically lowered levels of zinc in the blood occur in the course of infections with an accompanying strong inflammatory reaction. In the context of acute phase reaction, serum zinc shifts into the liver, which is mediated by the transport protein Zrt- and Irt-like protein 14 (Zip14). Moreover, it was shown that changes in zinc levels correlated with the severity and mortality rates of sepsis^12^. Data concerning zinc levels in patients with TAAA are sparse. In order to investigate the prognostic value of zinc levels before intervention, we retrospectively analyzed the clinical data available from 33 TAAA patients undergoing treatment. The impact of altered zinc levels on patients’ outcome after open and endovascular TAAA repair were assessed.

## Results

### Characteristics of the recruited patient cohort

We included 33 subjects undergoing TAAA repair (see Table 1). Our study population consisted of 17 female and 16 male patients. The age of the participating subjects ranged from 24 to 82 years with a mean of 63 years and a median of 66 years. The average body mass index (BMI) (kg/m^2^) was 25.4 (± 5.05; standard deviation (SD)). 14 patients underwent open surgical repair and 19 patients received endovascular intervention. Six patients suffered from connective tissue disease, namely Marfan syndrome, Loeyz-Dietz syndrome, alpha smooth muscle actin (*ACTA2*) mutation or suspected genetic aortic syndrome (GAS).

**Table 1:** Patients characteristics and procedural details.

Characteristics	All patients (n = 33)	Survivors		p-value
		Yes (n = 27)	No (n = 6)	
**Demographics**				
Age, years (mean ± SD)	63 ± 16.2	62.1 ± 16.6	67.2 ± 14.9	0.4986
Sex (female) (n)	51.52% (17)	51.85% (14)	50% (3)	0.9371
Body size, cm (mean ± SD)	173.6 (12.6)	173.7 (12.45)	173.2 (14.46)	0.9265
Weight, kg (mean ± SD)	76.89 (17.70)	80.61 (16.78)	60.17 (11.44)	0.0082
BMI, kg/m^2^ (mean ± SD)	25.4 (5.05)	26.6 (4.7)	20 (2.29)	0.0022
Smoking (current) (n)	36.36% (12)	33.33% (9)	50% (3)	0.4585
**Pre-existing conditions**				
Coronary heart disease (n)	42.42% (14)	44.44% (12)	33.33% (2)	0.6314
Peripheral arterial disease (n)	12.12% (4)	7.41% (2)	33.33% (2)	0.0829
COPD (n)	39.39% (13)	40.74% (11)	33.33% (2)	0.7465
Diabetes (n)	18.18% (6)	18.52% (5)	16.67% (1)	0.9185
GAS (N)	18.18% (6)	18.52% (5)	16.67% (1)	0.9185
Prior operations of the aorta (n)	48.49% (16)	93.75% (15)	6.25% (1)	0.0897
**Trace element levels before intervention (peripheral blood)**				
Zinc (mean ± SD)	57.52 (13.28)	60.09 (12.06)	45.92 (13.20)	0.0154
**Type of TAAA**				
TAAA 1 (n)	15.15% (5)	18.52% (5)	0% (0)	0.2664
TAAA 2 (n)	21.21% (7)	18.52% (5)	33.33% (2)	0.4379
TAAA 3 (n)	21.21% (7)	22.22% (6)	16.67% (1)	0.7721
TAAA 4 (n)	30.30% (10)	25.93% (7)	50% (3)	0.2595
TAAA 5 (n)	12.12% (4)	14.81% (4)	0% (0)	0.3298
**Procedure**				
Open intervention (n)	42.42% (14)	70.59% (12)	33.33% (2)	0.6314
Overall stay in hospital, days (mean ± SD)	29.06 (23.73)	31 (22.08)	22.5 (31.73)	0.4629
Duration of the intervention, min (mean ± SD)	374.30 (111.03)	356.52 (107.71)	454.33 (95.80)	0.0492
Total ventilation time, min (mean ± SD)	10,539.03 (25,818.35)	7807 (21,592.99)	22,831 (40,249.42)	0.2021
**Complications**				
Infections (n)	45.45% (15)	44.44% (12)	50% (3)	0.8120
Tracheotomy (n)	12.12% (4)	11.11% (3)	16.67 (1)	0.4910
MACE (n)	33.33% (11)	29.62% (8)	37.5% (3)	0.3539
AKI (n)	51.52% (17)	44.44% (12)	83.33% (5)	0.0897
Total number of red blood cell transfusions (mean ± SD)	13.76 (17.69)	9.44 (10.68)	33.17 (29.29)	0.0017
Total number of platelet transfusions (mean ± SD)	2.88 (3.71)	2.04 (2.81)	6.67 (5.09)	0.0039

Patients characteristics concerning demographics, pre-existing conditions, type of aneurysm, chosen procedure and complications. Shown are mean values ± standard deviation (SD) or percentage with total number in brackets.

*COPD* chronic-obstructive pulmonary disease, *TAAA* thoracoabdominal aortic aneurysm, *MACE* major adverse cardiac event, *AKI* acute kidney injury, *GAS* genetic aortic syndromes.

Significance was determined using Student's t-test assuming significance if *p < 0.05.

The average duration of the intervention was 374.30 min (± 111.03; SD). On average, patients remained in the hospital for 29.06 days (± 23.73; SD). Patients' intensive care stay was on average 7.61 days (± 10.99; SD)) long and during their hospital stay, patients were ventilated for 10,539.03 min (± 25,818.35; SD). 18.18% of all patients (n = 6) died during the inpatient stay. The average zinc level before interventions was 63.15 µg/dl for patients under 60 years compared to 55.07 µg/dl for patients that were 60 years or older. Moreover, there was a tendency for zinc levels to decrease comparably with the decline of renal function (Supplementary Fig. S1B).

It was also shown that decreasing BMI levels tended to be associated with decreasing serum zinc levels (Supplementary Fig. S1C; not significant). This was corroborated by the observation that decreased body weight correlated with a decrease in serum zinc levels (Supplementary Fig. S1D; p = 0.0285). Interestingly, body weight and BMI had also a significant (adverse) correlation with survival indicating that these patients were more likely suffering from a malnourished state (Table 1). Moreover, the duration of the intervention itself as well as the number of the administered blood products as red blood cells and platelets were significantly correlated to an increased rate of fatal outcomes (Table 1). We found no correlation between the zinc level and intake of different medications (e.g. antihypertensives, beta blockers, anticoagulants, diuretics or opiates) (Supplementary Fig. S2). Moreover, we found no correlation of serum zinc level and the presence of arterial hypertension, coronary heart disease (CHD), chronic obstructive pulmonary disease (COPD), diabetes or previous aortic intervention (Supplementary Fig. S2). In addition, we do not see any association between blood zinc concentrations and the smoking behavior, the presence of allergy, the occurrence of major adverse cardiac events (MACE) or even the occurrence of acute kidney injury (AKI) during treatment (Supplementary Fig. S3).

### Low serum zinc levels before intervention correlate with increased mortality

**Figure 1:**
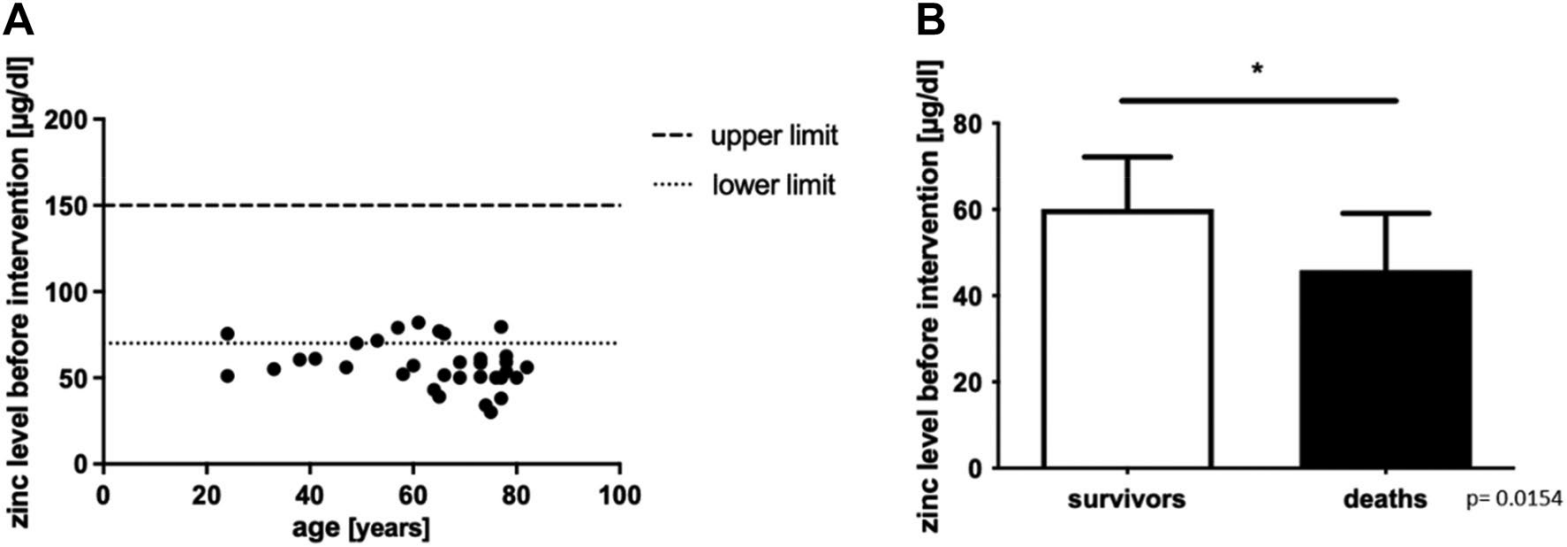
(**A**) Zinc levels of all patients (n = 33) in correlation with the age before endovascular/surgical intervention are shown. The dashed lines show commonly used upper (70 µg/dl)/lower (150 µg/dl) standard values for the zinc level. (**B**) The zinc levels of patients that survived endovascular/surgical intervention and were released from the hospital (n = 27) were compared to patients that died during the hospital stay (n = 6). Zinc level was measured before endovascular/surgical intervention. Shown is the mean and SD. Significance was determined using Student's t-test assuming significance if *p < 0.05.

**Figure 2:**
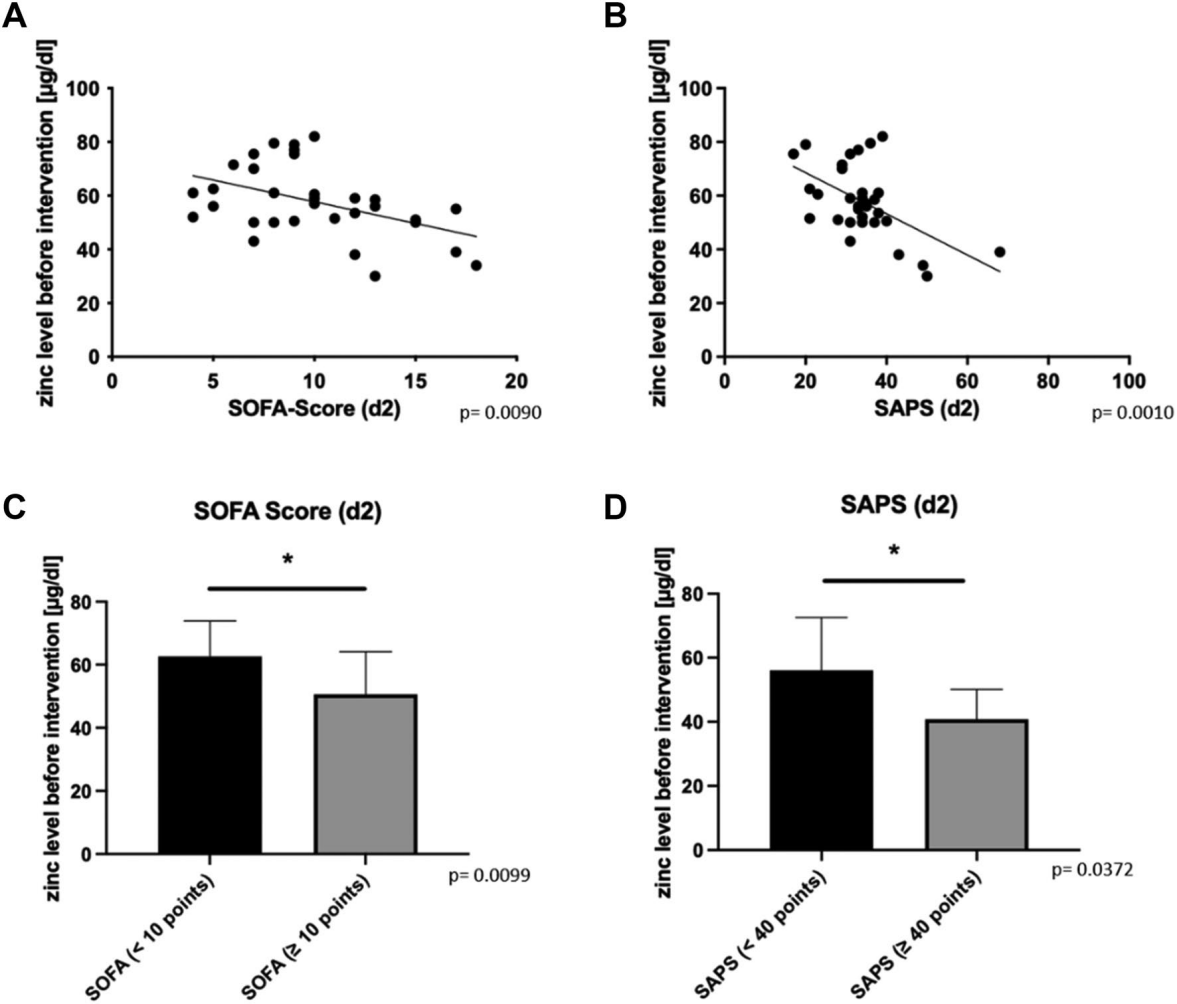
(**A**) The SOFA-Score (Sepsis-related organ failure assessment) of 32 patients after two days is shown. For one patient no SOFA-score could be obtained after two days. Accompanying the regression line (p = 0.0090). (**B**) The SAPS (Simplified Acute Physiology Score) for 32 patients after two days is demonstrated. For one patient no SAPS could be obtained on day two. Also shown is the regression line (p = 0.0010). (**C**) Demonstrated is the SOFA score after two days according to the point value < 10 points (n = 19) or ≥ 10 points (n = 13) (0.0099). (**D**) Shown is the SAPS score divided into patients with a score < 40 points (n = 26) or patients with ≥ 40 points (n = 6) (p = 0.0372).

A large proportion of patients showed a serum zinc level below 70 µg/dl, which was used as cut-off value for incipient zinc deficiency (Fig. 1A)^13^. As reference values, we used 70 µg/dl zinc as the lower limit and 150 µg/dl as the upper limit. These limit values are frequently reported as part of routine diagnostics^13^. Patients who had decreased zinc levels before intervention showed an increased in-hospital mortality rate (Fig. 1B; p = 0.0154). At the time before intervention, there was no correlation between the value of C-reactive protein (CRP) compared with the measured zinc levels. Patients with an initial CRP value under 10 mg/l (n = 11) had a mean zinc level before the intervention of 59.26 µg/dl compared to 53.91 µg/dl in patients with an CRP value above 10 mg/l (n = 21) (not significant; p = 0.2934). The prediction of worse outcome by the zinc level before intervention is strengthened by the fact that lowered zinc levels were associated with worse intensive care risk scores, namely SOFA-Score and SAPS. Patients with decreased zinc levels at baseline showed a higher ranking in SOFA-Score on day 2 (Fig. 2A) as well as in SAPS on day 2 (Fig. 2B). Patients with a SOFA-Score on day 2 above 10 points (n = 13) had an average zinc level of 50.58 µg/dl before the intervention compared to 62.66 µg/dl for patients that showed lower values in the SOFA-Score (n = 19) (Fig. 2C; p = 0.0099). It is particularly interesting that we see this correlation for zinc levels before the respective intervention. For the SAPS score, comparable findings were observed. Patients with a SAPS above 40 points on day 2 had an initial zinc level about 40.83 µg/dl (n = 6) compared to 56.12 µg/dl (n = 26) in patients with a better prognostic assessment using SAPS (Fig. 2D; p = 0.0372).

### Zinc level and the acute phase reaction

It has previously been shown that zinc levels in serum decrease during the acute phase reaction due to a physiological zinc shift. In our study we could not find a significant correlation between zinc levels and inflammatory parameters. Zinc levels 24 h (Supplementary Fig. S4A) and 7 days (Supplementary Fig. S4B) after intervention showed no significant correlation with CRP levels on the same day, respectively. Even immediately after intervention or 24 h after, the comparison of zinc levels with the CRP levels of the coming days, showed no significant correlation. Nevertheless, a tendency of decreased zinc levels concomitantly to an increase of the CRP values over the course of the following days was observed. We demonstrate that patients with higher zinc levels after intervention tended to have lower CRP values on day 2 (Supplementary Fig. S4C; p = 0.0642). Comparable results were observed for the zinc level after 24 h and the CRP level on day 3 (Supplementary Fig. S4D; p = 0.1179). Since it is only possible to detect an increase in the CRP after several hours^14^ and only one measurement per day was possible, we consider the approach to be justified. Although the correlation between zinc level and inflammation has been described previously by various publications^15–18^, we could not demonstrate a correlation in our patient collective. Likewise, we did not observe any correlation with other infection parameters such as procalcitonin (PCT) or interleukin (IL)-6 (data not shown).

### Serum zinc level and infection

Zinc levels 12 h after intervention were not significantly decreased in patients who experienced septic shock during the course of treatment (Supplementary Fig. S5A). The same is true for the difference of zinc (delta zinc level) 12 h after intervention compared to the zinc level at the time of inpatient admission (Supplementary Fig. S5B). Patients who developed pneumonia, urinary tract infection (UTI) or both during the course of the study did not show lower zinc levels after 12 h compared to patients for whom no specific infection was documented (Supplementary Fig. S5C). In addition, for patients with pneumonia, UTI or both we could not observe an augmented serum hypozincemia 12 h after the intervention (Supplementary Fig. S5D).

### Chronic inflammation and atherosclerosis

**Figure 3:**
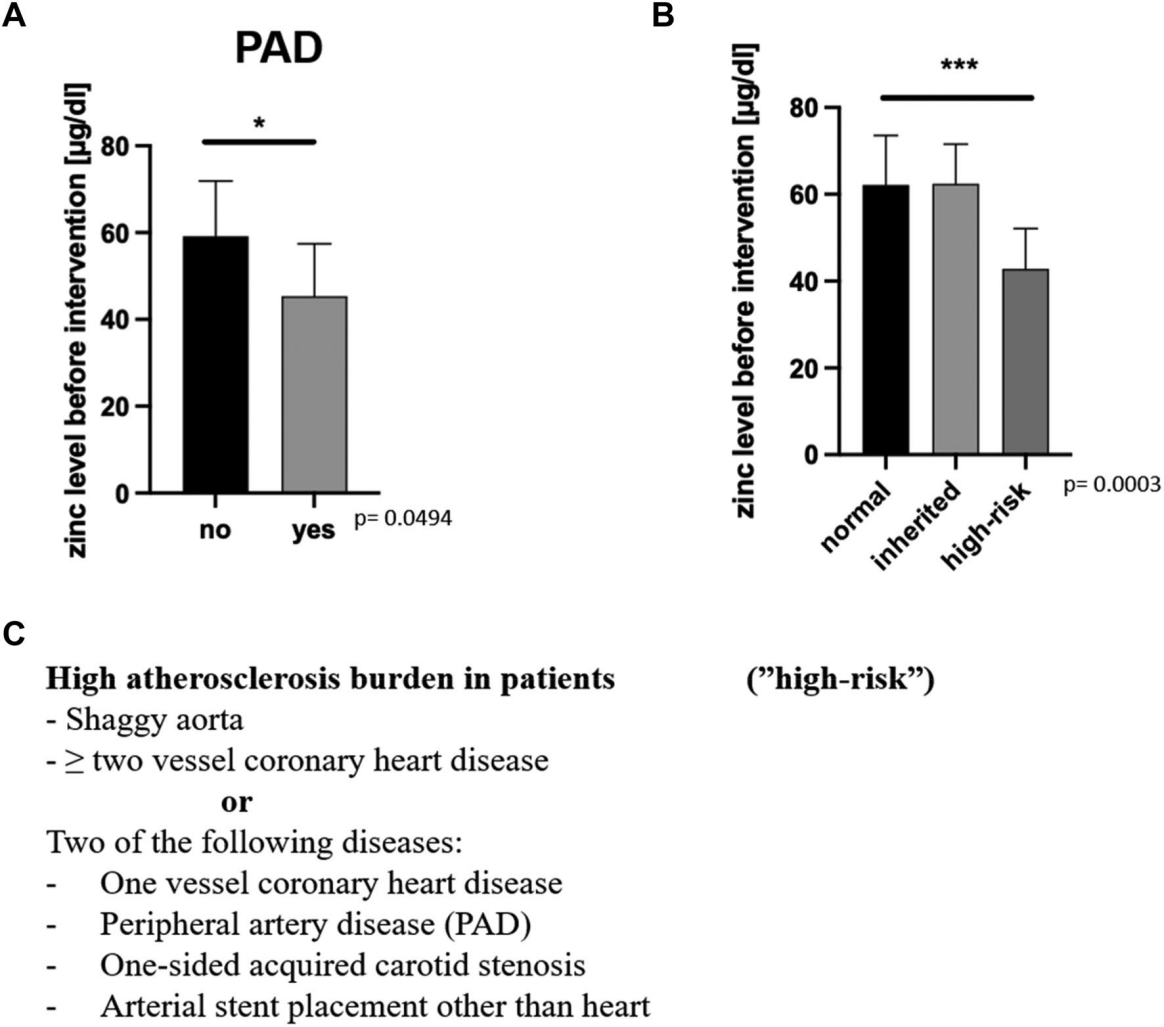
(**A**) Zinc levels before intervention of patients who had or did not have peripheral arterial disease (“PAD”; n = 4; p = 0.0494). (**B**) Mean zinc level of our patient cohort before the respective intervention depending on whether we assigned the patients to the group with proven (n = 5) or high suspicion (n = 1) of a genetic predisposition (“inherited”; n = 6; p = 0.9601) or to the group with high atherosclerosis burden ("high-risk"; n = 8; p = 0.0003). (**C**) Representation of the criteria that we used to categorize patients to the high-risk group (“high-risk”) or not (“normal”). Shown is the mean and SD. Significance was determined using Student's t-test assuming significance if *p < 0.05, **p < 0.01 and ***p < 0.001.

The correlation of zinc deficiency with atherosclerosis is subject of an ongoing debate^19–22^. We did not observe a correlation between coronary artery disease and zinc level in our patient population. However, we showed that patients with PAD had significantly lower zinc levels at the time of admission (Fig. 3A). Since PAD is typically caused by atherosclerotic plaques, a correlation of the serum zinc level with this atherosclerotic disease is likely. This was the motivation to classify patients based on their degree of atherosclerosis-related diseases. Furthermore, due to the fact that both the consecutive occurrence of new atherosclerotic lesions^23^ and the number of manifestation sites in the human body partially reflect the burden of atherosclerosis^24^, we have graded the severity of atherosclerosis in our patients based on the available data. In the presence of a shaggy aorta or in the case of atherosclerosis at two different sites, the patients were classified into a "high-risk" group. The high-risk group represents patients with elevated risk for atherosclerosis-related life-threatening complications (Fig. 3C)^24^. We did not observe a difference between patients with a normal-risk score (n = 19) and patients who have a congenital cause for aneurysms (n = 6) (Fig. 3B). Our patient cohort included patients with a familial predisposition to aneurysm formation (n = 6). Three patients were found to have Marfan syndrome, one patient had Loeyz-Dietz syndrome, one patient had an *alpha smooth muscle actin* (*ACTA2*) mutation and one patient showed a suspected congenital disorder because of various aneurysms in the patient’s medical history. Patients that we classified as high atherosclerotic burden (“high risk”; n = 8) showed significantly decreased zinc levels at the time of hospital admission (Fig. 3B). This is in line with the decreased zinc level in patients with PAD (Fig. 3A) and supports the assumption of lowered serum zinc levels in patients with degenerative vascular diseases.

## Discussion

In our study, decreased serum zinc levels in patients undergoing TAAA repair were found to be related to lower survival rates (Fig. 1B) and correspondingly worsened intensive care prognostic scores two days after intervention (Fig. 2A,B). Our study cohort of 33 patients, although all have TAAA, presents itself as quite small and heterogeneous group (Table 1). Impressively, a large proportion of patients showed decreased serum zinc levels before surgery, which can be interpreted as a concomitant effect of pre-existing diseases and an altered nutritional status (Fig. 1A). Moreover, there was no association observed between preoperative zinc levels and most patients’ comorbidities (like hypertension, coronary heart disease, COPD, diabetes mellitus), medication (antihypertensives, beta-blockers, anticoagulants, diuretics and opiates) or prior operations of the aorta (Supplementary Fig. S2). We were able to confirm previous observations, such as the negative (not significant) association of serum zinc level with age^25^ (Supplementary Fig. S1A) as well as the negative (not significant) correlation with renal dysfunction^26^ (Supplementary Fig. S1B). However, some publications do not indicate a correlation of zinc level with age and they explain the differences in serum zinc levels by the existence of other comorbidities^27^. By the fact that our patients’ body weight and BMI correlates significantly with survival, we believe that a large proportion of patients with severe zinc deficiency are malnourished and have a nutrition-induced zinc deficiency^28,29^. Nevertheless, the preoperative low zinc level had an impact on mortality, severity of the post-interventional intensive care scores, as well as the prevalence of atherosclerosis-related diseases in TAAA patients. A large proportion of the patients studied showed serological zinc deficiency which is consistent with the suggestion that even in the western world many people are affected by zinc deficiency with predominance of older people and patients with chronic diseases among those affected. So far, a clear relationship between atherosclerosis and zinc deficiency has not been proven with certainty^22,30^, but other studies suggested a correlation between low zinc levels and atherosclerotic diseases such as an association between zinc deficiency and coronary heart disease^31^. Previously published studies underlined that zinc deficiency appears to promote the production of proinflammatory cytokines such as IL-1β, tumor necrosis factor alpha (TNFα), and IL-6 by myeloid cells and activated monocytes/macrophages^32,33^. Zinc supplementation of elderly people suffering from zinc deficiency was able to improve immune cell function and abnormal cytokine expression^32,33^. Despite the described correlation of a low zinc level with the acute phase response^33^, we were not able to show this relationship in our cohort (Supplementary Fig. S4). We also observed comparable effects on patient prognosis. Furthermore, it was previously shown that zinc deficiency is associated with increased number of proinflammatory cytokines and increased activation of proinflammatory signaling cascades^33,34^. At least 50% of our patients who died had concomitant infections as diagnosis and at least one out of six persons who died had a clinically relevant impairment of wound healing. It should be pointed out once again that zinc has an significant impact on both immune function^35^ as well as wound healing^36^. Since there are studies showing that zinc supplementation can reduce the excessive release of proinflammatory cytokines in elderly individuals, the possible supplementation of the trace element zinc could be considered^37^. As a perspective, further studies should clarify whether zinc supplementation can reduce the number of inflammatory cytokines, the progression of atherosclerosis and the occurrence of deadly adverse events in patients with TAAA. We hypothesize that patients who enter treatment with already markedly decreased zinc levels suffer from worse zinc deficiency in the peripheral blood due to acute phase reaction during therapy leading to increased patient mortality.

The heterogeneous character of the assessed, rather small cohort of patients in this retrospective study is a relevant risk of bias and has to be mentioned as a relevant limitation. In addition, the data evaluation of zinc levels after intervention is problematic because critically ill patients also received artificial diets containing zinc chloride as part of intensive care treatment. Unfortunately, it is not possible to retrospectively work out the dose of zinc received in each case. This is most likely another reason why we could not observe a correlation between follow-up zinc level as well as zinc shift concerning infectious complication, changes in inflammatory values and wound healing disorders. We would like to emphasize the observational character of our study, but at the same time we underlined potential importance of zinc levels in TAAA patients. We wish that our study will give impetus to new mechanistic studies regarding the micronutrient zinc and its impact on the survival of patients with atherosclerosis-related diseases.

## Conclusion

Patients with fatal outcome after TAAA repair showed reduced pre-interventional zinc levels. Zinc deficiency represents a potential phenomenon of chronic inflammation especially in patients with severe atherosclerosis, which is a common comorbidity of TAAA patients. We confirm decreased serum zinc in patients with atherosclerotic PAD and also in patients with a high burden of atherosclerosis. We speculate that decreased zinc levels before intervention may be related to a poorer outcome because of negatively altered perioperative inflammation reaction. Therefore, a preoperative screening of the zinc status could be considered for patients undergoing TAAA repair.

## Materials and methods

### Study population

In this retrospective observational study 33 patients suffering from TAAA requiring treatment because of a diameter above 5.5 cm or in case of connective-tissue disease, above 5.0 cm. Patients were included between the 11th of January and the 20th of December 2017. All patients were treated at the RWTH Aachen University Hospital. Patients that underwent an open or endovascular TAAA repair wereevaluated. Exclusion criteria were the following: age below 18 years, pregnancy, patients with immunosuppressive medication and patients with pre-existing need for renal replacement therapy. Moreover, no emergency interventions were included.

Relevant comorbidities such as chronic-obstructive pulmonary disease (COPD), coronary heart disease and diabetes, all defined according to current guidelines^38–40^, pre-existing medication included antihypertensives, beta blockers, anticoagulants, diuretics or opiates were correlated to patients’ zinc levels. Genetic Aortic Syndromes (GAS), namely Marfan syndrome, Loeyz-Dietz syndrome, alpha smooth muscle actin (*ACTA2*) mutation and suspected genetic aortic syndrome were assessed. All patients with peripheral arterial disease (PAD) showed disease stage II b or higher according to the Fontaine classification^41^.

Extensive atherosclerosis or extensive thrombus load of the aorta, also called "shaggy aorta", has been described to be an impressive sign of systemic atherosclerosis^42^.

Physiological parameters and patients’ medical history namely c-reactive protein (CRP), procalcitonin (PCT) and interleukin-6 were taken from the electronic medical records (IntelliSpace Critical Care and Anesthesia; Philips Healthcare, Andover, Massachusetts, USA). The sequential organ failure assessment (SOFA)^43^ and the simplified acute physiology score (SAPS) were assessed at different time points^44^. During the course of therapy, the SOFA and the SAPS were performed on days 1 (SOFA: n = 14; SAPS: n = 5), 2 (SOFA: n = 32; SAPS: n = 32), 3 (SOFA: n = 29; SAPS: n = 29), 7 (SOFA: n = 15; SAPS: n = 15), 14 (SOFA: n = 8; SAPS: n = 8) and 28 (SOFA: n = 4; SAPS: n = 4). For both scores, an increased amount of score points indicate a reduced general condition of the patient as well as a worse prognosis with higher probability of in-hospital mortality. Acute kidney injury (AKI) within 48 h postoperatively was defined according to the Kidney Disease Improving Global Outcomes (KDIGO) criteria^45^.

Major cardiovascular events (MACE) included acute heart failure, myocardial infarction and ventricular tachycardia were defined according to current guidelines^46–48^. Sepsis was defined according to the German Sepsis Society^49^. The category “infections” included pneumonia, urinary tract infection and surgical site infection^50–52^. All patients underwent informed consent and agreed to participate in the research project. The study protocol was approved by the ethic committee of the University Hospital RWTH Aachen, Germany (EK 004/14) and was conducted in accordance with the ethical standards laid down in the Declaration of Helsinki. Prior to this, other studies were also conducted based on this patient cohort^53–55^.

### Acquisition of the patient material

Serum tubes (SARSTEDT S monovette) were collected from the patients at different time points. Samples were collected before intervention, after admission to the intensive care unit (ICU) (direct after intervention), as well as during ICU follow-up (12 h, 24 h, 48 h, and 7 days). All patients had a study-related blood draw before the intervention (n = 33). Blood sampling as postoperative follow-up did not occur in all patients at respective time points. Acquisition of patient blood serum occurred immediately after the intervention in n = 30 patients, after 12 h in n = 32, after 24 h in n = 31 patients, after 48 h in n = 30 patients, and after 7 days in n = 24 patients. The material was preserved at − 20 °C until further processing. Although the Sarstedt tubes used are not specially designed for the measurement of trace elements like zinc, both external work^56^ as well as internal validations in comparison to special sample tubes designed for trace element measurements show that same zinc levels were detected independently of the used test tube (data not shown).

### Measurement of the total serum zinc level

The serum zinc concentration was determined by flame Atomic Absorption Spectrometry (AAS) using an AAnalyst 800 (Perkin-Elmer, Waltham, United States). For measurement serum samples were diluted as described elsewhere^57,58^.

### Surgical intervention

As published before, the protocol for open TAAA repair included aortic cross-clamping, extracorporal circulation with distal aortic perfusion, and visceral perfusion using selective perfusion catheters^59,60^. To avoid acute renal failure, contrast agent was used carefully, leading to a mean application of 65 ± 17 ml per endovascular procedure. Furthermore, we applied one fourth of the standard dose for kidney angiography^61^.

### Classification of atherosclerosis severity

In the presence of atherosclerosis-related diseases, we classified patients into a high-risk category as soon as they had more than one severe manifestation of atherosclerosis (see Fig. 3C). By doing so, we invoke data suggesting that respective disease severity and the amount of manifestations likely reflect the level of atherosclerotic disease burden^23,24^. As an exception, we consider the presence of a shaggy aorta as a high-risk criteria because itself represents an extensive, severe, and rather rare arteriosclerosis-related disease of the whole aorta^42^.

### Statistics

Statistical significances were calculated by Student’s t test using GraphPad Prism software (version 5.01). Significances are indicated by: *p < 0.05, **p < 0.01 and ***p < 0.001. For linear regression we calculated the F test and we assume a significant deviation from zero if *p < 0.05, **p < 0.01 and ***p < 0.001.

### Institutional review board statement

The study was conducted according to the guidelines of the Declaration of Helsinki, and approved by the Institutional Review Board of the University Hospital RWTH Aachen EK010/19, 21th May 2019.

### Informed consent

Informed consent was obtained from all subjects involved in the study.

## Supplementary Information

Supplementary Information.

## Data Availability

Data available on request from the authors.
