# Supplementary material for: Retrospective observational study evaluating zinc plasma level in patients undergoing thoracoabdominal aortic aneurysm repair and its correlation with outcome
Source: Sci Rep. 2021 Dec 21;11:24348. doi: 10.1038/s41598-021-03877-6 (PMC8692510; doi:10.1038/s41598-021-03877-6)
Supplement: Supplementary file 1 — Supplementary Information. [file 41598_2021_3877_MOESM1_ESM.pdf]

**Supplementary Figure 1:**

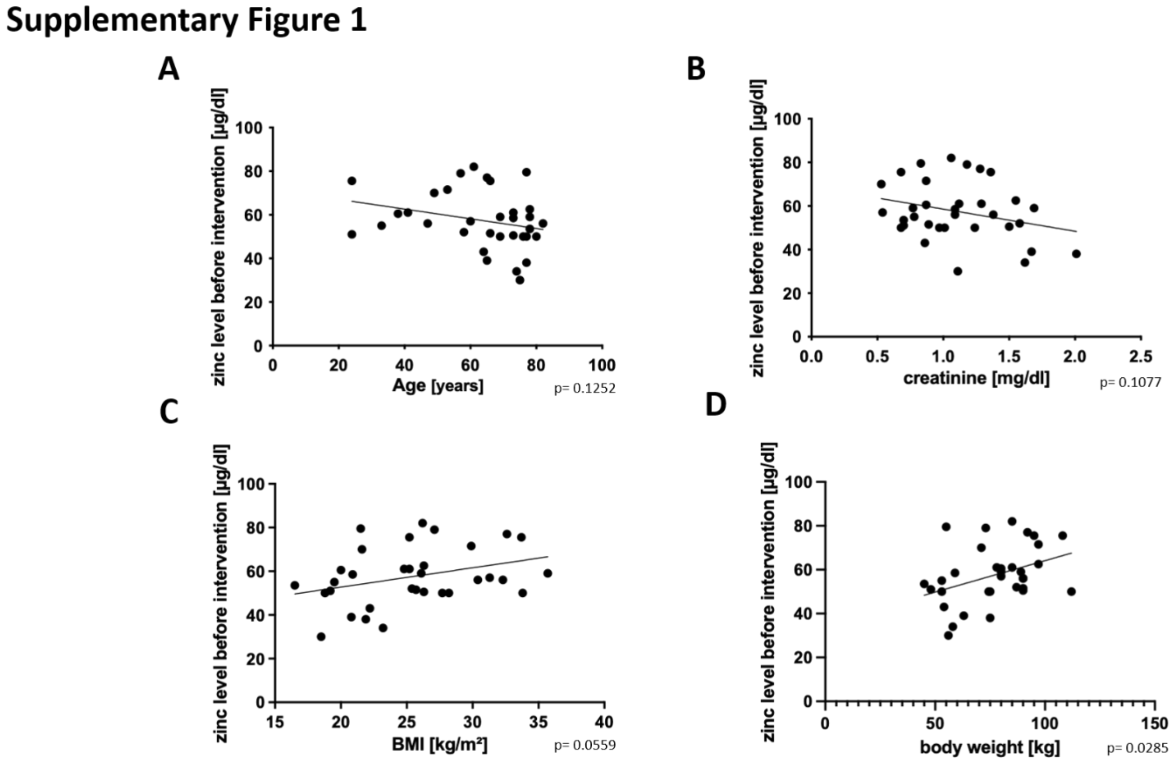


**A)** Zinc levels of all patients in correlation to the age before intervention (n=33) are shown. The regression line was calculated from all measured values (p=0.1252). **B)** Zinc levels before intervention in correlation to the creatinine value at the time of inpatient admission (n=33) with the corresponding regression line (p=0.1077). **C)** The graphic shows the zinc level before intervention in correlation to the body mass index (BMI) (n=33) with the corresponding regression line (p=0.0559). **D)** Zinc levels before intervention in correlation to the body weight (n=33) together with the corresponding regression line (p=0.0285) are demonstrated.

**Supplementary Figure 2:**

## Supplementary Figure 2

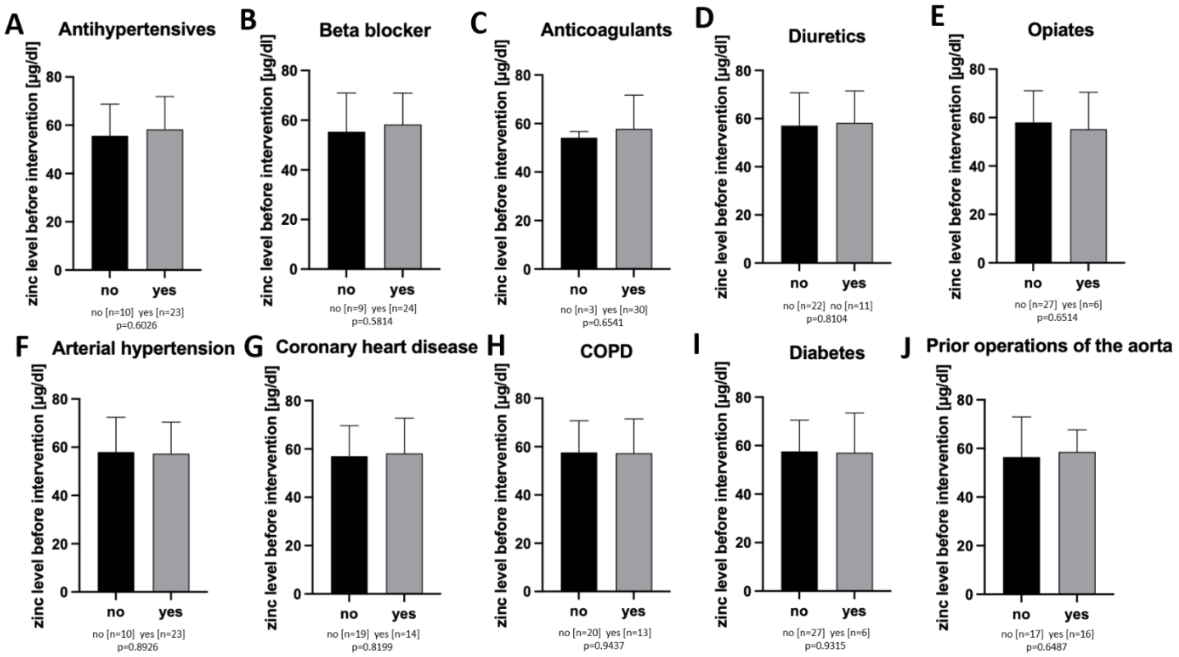


Individual medications, such as antihypertensives (**A**), beta blockers (**B**), anticoagulants (**C**), diuretics (**D**), and opiates (**E**), and their relationship to zinc level before intervention are shown. Also shown is the zinc level before intervention in the presence of arterial hypertension (**F**), coronary heart disease (**G**), chronic obstructive pulmonary disease (COPD) (**H**), diabetes (**I**), or prior operations of the aorta (**J**). The data were available for all patients (n=33).

**Supplementary Figure 3:**

## Supplementary Figure 3

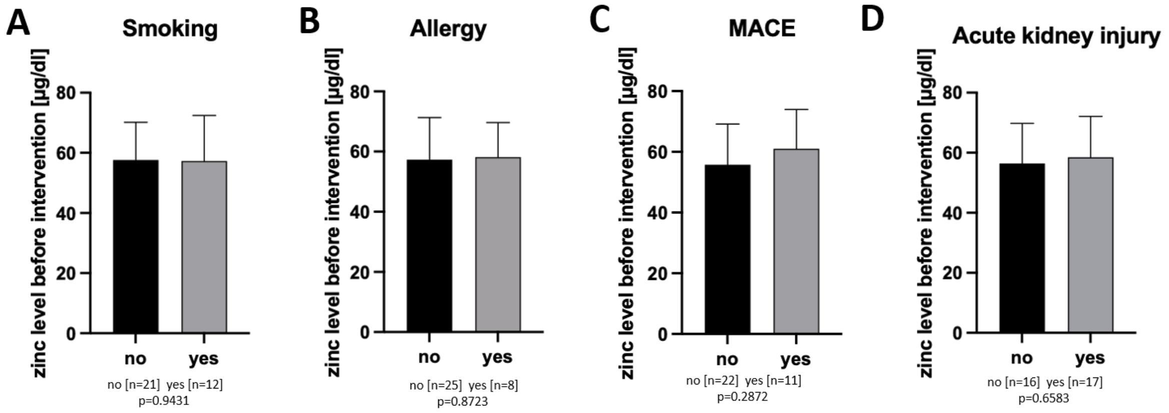


Relationship of pre-intervention zinc level with patient smoking behavior (**A**), presence of allergies (**B**), the incidence of major adverse cardiac events (MACE) during inpatient treatment (**C**), and the occurrence of acute kidney injury (AKI) (**D**). The data were available for all patients (n=33).

**Supplementary Figure 4:**

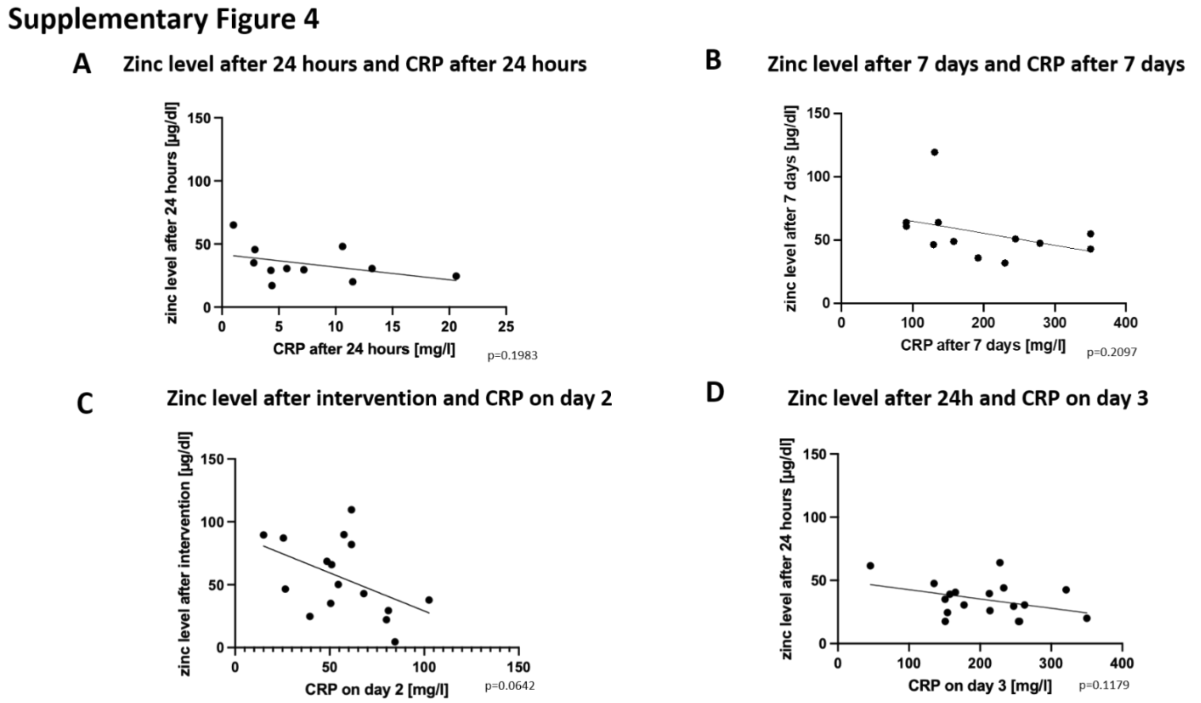


The relationship between zinc level and concentration of C-reactive protein (CRP) in the peripheral blood is shown. **A)** Relationship between zinc level after 24 hours and CRP level after 24 hours (n=11; p=0.1983). **B)** Relationship between zinc level after 7 days and CRP level after 7 days (n=12; p=0.2097). **C)** Zinc value immediately after the intervention and the CRP value after two days (n=16; p=0.0642). **D)** Zinc value after 24 hours intervention and the CRP value after three days (n=18; p=0.1179).

**<u>Supplementary Figure 5:</u>**

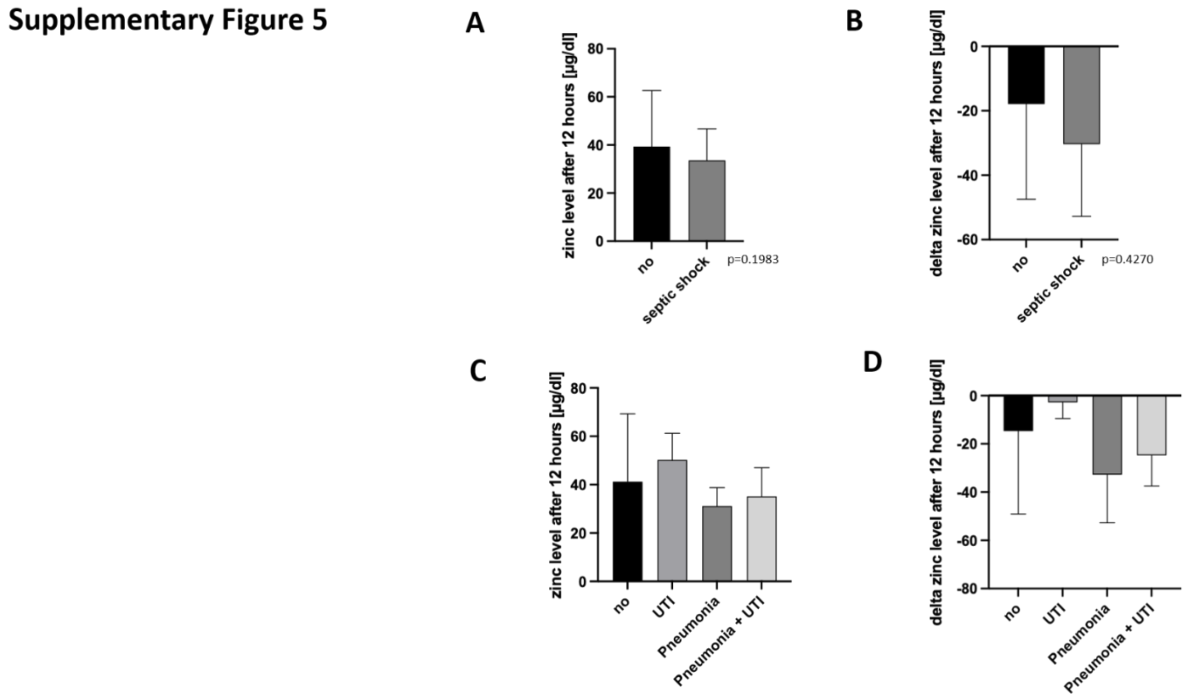


**A)** Zinc levels 12 hours after intervention of patients who have not developed a septic shock (“no”) or who have developed septic shock in the course of treatment (“septic shock”; n=4; p=0.6378). **B)** Delta zinc between the zinc level before intervention and 12 hours after depending on whether septic shock has occurred in the course of treatment (“septic shock”; n=4; p=0.4270) or not (“no”). **C)** Zinc level 12 hours after intervention depending on

whether a specific infectious complication was reported for the patient such as urinary tract infection (“UTI”; n=2), pneumonia (“Pneumonia”; n=7; p=0.3642) or both infections either concurrently or sequentially (“Pneumonia + UTI”; n=5; p=0.6434). **D)** Delta zinc between the zinc level before intervention and 12 hours after depending on whether a specific infectious complication was reported for the patient such as urinary tract infection (“UTI”; n=2), pneumonia (“Pneumonia”; n=7; p=0.2078) or both infections either concurrently or sequentially (“Pneumonia + UTI”; n=5; p=0.5329). Shown in each case is mean and SD.
